# Elevated Plasma Stromal-Cell-Derived Factor-1 Protein Levels Correlate with Severity in Patients with Community-Acquired Pneumonia

**DOI:** 10.1155/2014/829706

**Published:** 2014-10-13

**Authors:** Ping-Kun Tsai, Ming-Ju Hsieh, Hsiang-Ling Wang, Ming-Chih Chou, Shun-Fa Yang, Chao-Bin Yeh

**Affiliations:** ^1^Department of Internal Medicine, Zuoying Branch of Kaohsiung Armed Forces General Hospital, Kaohsiung 813, Taiwan; ^2^Department of Emergency Medicine, Chung Shan Medical University Hospital, Taichung 402, Taiwan; ^3^Institute of Medicine, Chung Shan Medical University, No. 110, Section 1, Chien-Kuo N. Road, Taichung 402, Taiwan; ^4^Cancer Research Center, Changhua Christian Hospital, Changhua 500, Taiwan; ^5^Department of Beauty Science, National Taichung University of Science and Technology, Taichung 403, Taiwan; ^6^School of Medicine, Chung Shan Medical University, Taichung 402, Taiwan; ^7^Department of Medical Research, Chung Shan Medical University Hospital, Taichung 402, Taiwan; ^8^Department of Emergency Medicine, School of Medicine, Chung Shan Medical University, Taichung 402, Taiwan

## Abstract

*Background.* The aim of this study was to investigate differential changes in plasma levels of stromal-cell-derived factor-1 (SDF-1) before and after antibiotic treatment in patients with community-acquired pneumonia (CAP) and observe the association between the severity of CAP and the plasma SDF-1 level. *Methods.* We gathered blood specimens from 61 adult CAP patients before and after antibiotic treatment and from 60 healthy controls to measure the plasma concentrations of SDF-1 by using an enzyme-linked immunosorbent assay. *Results.* The plasma SDF-1 concentration was elevated significantly in patients with CAP before receiving treatment compared with the controls and decreased significantly after the patients received treatment. Leukocyte (WBC) and neutrophil counts and C-reactive protein (CRP) levels decreased significantly after antibiotic treatment. Moreover, differences in the plasma concentration of SDF-1 were significantly correlated with PSI, CURB-65, and APACHE II scores (*r* = 0.389, *P* = 0.002, and *n* = 61; *r* = 0.449, *P* < 0.001, and *n* = 61; and *r* = 0.363, *P* = 0.004, and *n* = 61, resp.). *Conclusions.* An elevated plasma SDF-1 concentration can be used as a biological marker for the early diagnosis of CAP and for the early detection of its severity.

## 1. Introduction

Community-acquired pneumonia (CAP) is pneumonia that is not acquired in a hospital or a long-term care facility within the past 14 days [1]. In the United States, the total cost of health care for CAP was $8.4 billion in 2001, and 5.6 million cases of CAP occur each year [1, 2]. The combination of pneumonia and influenza is the eighth leading cause of death in the United States [3]. In Taiwan, pneumonia was the fourth leading cause of death in 2012, according to the statistics of the Ministry of Health and Welfare [4]. Therefore, diagnosing and treating CAP early is vital to reducing morbidity and mortality [3]. Clinically, the leukocyte (WBC) count and C-reactive protein (CRP) level are used to monitor pneumonia severity [5]. However, several studies have questioned using the WBC count and CRP to predict the prognosis of CAP [6, 7]. The specificity and sensitivity of these diagnostic markers are not good enough, especially for predicting CAP severity. Therefore, the purpose of this study was to investigate the biological markers for early diagnosis and detect the severity of CAP.

The Pneumonia Severity Index (PSI) is used worldwide, including Taiwan. Physicians determine the disposition of CAP patients by evaluating the severity of CAP according to the PSI. The CURB-65 score, which is the sum of 5 risk factors (i.e., confusion, urea, respiratory rate, blood pressure, and an age of 65 years or older), measures the severity of CAP [8, 9]. Patients scoring 0, 1, and 2 according to CURB-65 have a 30-day mortality of 0.7%, 3.2%, and 3%, respectively. One study reported that the PSI and CURB-65 scoring systems were similar in predicting the 28-day in-hospital mortality of the patients with severe sepsis and CAP [10].

SDF-1, also called CXCL12, is a chemotactic cytokine belonging to the large family of CXC chemokines. SDF-1 is related to a different chemokine-chemokine receptor axis and regulates the movement of neutrophils, monocytes, T-lymphocytes, and basophils. SDF-1 also induces cell migration, cell adhesion, neutrophil activation, and inflammation [11]. Another study reported that the CXCR4/SDF-1 axis plays a crucial role in the recruitment of neutrophils to the lung during acute lung injury, and this cytokine axis was noted in the reparative response to lung injury [12]. SDF-1 signaling during sepsis is vital for neutrophil bone marrow mobilization and host survival [13]. Overexpression of SDF-1 has been reported to be associated with inflammatory diseases, such as rheumatoid arthritis (RA), acute myocardial infarction, pelvic inflammatory disease (PID), and pathogenesis of atherosclerosis [14–16] as well. Furthermore, neutrophils and T-lymphocytes are abundant in the inflammatory lesions of patients with pneumonia and a high neutrophil cell count is found in patient's blood [5]. Thus, we hypothesized that the expression of SDF-1 protein is associated with CAP. Although several functions of SDF-1 have been reported, no study has investigated the prognostic value of SDF-1 in a cohort of patients with CAP or proved the association between the severity of CAP and SDF-1. In this study, we measured the plasma levels of the SDF-1 protein in a group of patients with CAP and in healthy control participants to evaluate whether SDF-1 is a useful biochemical marker to differentiate between healthy people and patients with pulmonary infectious disease.

## 2. Materials and Methods

### 2.1. Participants and Diagnosis

This study enrolled 121 people (61 CAP patients and 60 healthy controls) from February 2009 to December 2009 at Chung Shan Medical University, Taichung, Taiwan. For a control group, who visited the Department of Family and Community Medicine for health examination in Chung Shan Medical University Hospital, were selected as healthy controls. This study was approved by the Chung Shan Medical University Hospital Institutional Review Board. Demographic characteristics, comorbidities, symptoms and signs of pneumonia, laboratory results, and the previous antibiotic treatments of each patient were recorded upon admission. The guidelines of the Infectious Diseases Society of America/American Thoracic Society were used as diagnostic criteria [17]. The criteria for CAP diagnosis were a typical infiltration change on chest X-ray films within 1 day of symptom occurrence and at least one clinical manifestation, such as cough, yellow and thick sputum, or high fever (>37.8°C), or at least 2 minor criteria, including tachypnea, dyspnea, pleural pain, chest pain, confusion or disorientation, lung consolidation, or WBC counts > 12000 cells/*μ*L. Exclusion criteria were outpatient status, transfer from another hospital or hospital admission within the previous 3 weeks, the presence of other acute conditions such as pulmonary edema, pulmonary embolism, or malignancy appearing during the follow-up period, pneumonia caused by tuberculosis or malignancy, and severe immunocompromisations, including severe neutropenia (WBC count lower than 1.0 × 10^9^ cells/L), organ or bone marrow transplant, and HIV infection. Moreover, intake of anti-inflammatory drugs like corticosteroids was also excluded. The pneumonia severity indices were assessed using the PSI, Acute Physiology and Chronic Health Evaluation II (APACHE II), and CURB-65 tests [18–20].

### 2.2. Blood Specimen Collection

The blood samples from all patients with CAP were obtained to test the WBC and neutrophil cell counts, CRP concentration, and plasma concentration of SDF-1 before and after antibiotic treatment. Blood samples of the control group were also collected and tested. The samples were placed in tubes containing EDTA, and centrifugation was immediately performed. The samples were stored at −80°C. Patients with CAP received treatment with antibiotics such as cefuroxime, ceftizoxime, and clarithromycin according to their condition.  Table 1 presents a summary of clinical data and the demographics of the patients and controls.

### 2.3. Measurement of the Plasma SDF-1 Level Using an Enzyme-Linked Immunosorbent Assay

An enzyme-linked immunosorbent assay (ELISA) was used to measure the plasma concentrations of SDF-1 in all blood samples (Quidel Corporation, San Diego, USA). Each plasma sample (100 *μ*L) was directly transferred to the microtest strip wells of the ELISA plate and subsequently incubated for 1 hour at room temperature. After 4 washing steps, the detection antibody was added, and the reaction system was incubated for 1 hour at room temperature. Antibody binding was detected using streptavidin-conjugated horseradish peroxidase and developed using a substrate solution. The reaction was then stopped, and the optical density was determined using a microplate reader set at 450 nm. Soluble SDF-1 concentrations were quantitated according to a calibration curve using a human SDF-1 standard. Each plasma sample was assayed according to the manufacturer's instructions, and the values were within the linear portion of the standard curve.

### 2.4. Statistical Analysis

SPSS 15.0 statistics software (SPSS Inc., Chicago, IL) was used to perform statistical analysis. All continuous variables were expressed as mean ± SD. The Mann-Whitney *U* test was used to compare the differences between untreated patients and healthy controls in continuous variables that did not follow a parametric distribution. The Wilcoxon signed-rank test was used to compare the differences in categorical variables between the untreated and treated patients. A Pearson correlation coefficient assessed the association of SDF-1 levels relative to the laboratory variables of patients with CAP. We then plotted receiver-operating characteristic curves (ROCs) to select the cutoff levels of plasma SDF-1 to distinguish patients with pneumonia from normal individuals. Sensitivity, specificity, positive predictive value, and negative predictive value were calculated. Statistical significance was defined as *P* < 0.05 in 2-tailed tests.

## 3. Results

A summary of the demographic and clinical characteristics of the participants is presented in Table 1. The analysis in this study was based on a sample of 121 people. The age and sex of the participants did not significantly differ between the CAP group and the control group. According to the PSI, 39, 18, and 4 patients with CAP were classified as low risk, moderate risk, and high risk, respectively. According to CURB-65, 26, 17, and 18 patients were assigned scores 0, 1, and 2, respectively. In addition, 48 and 13 patients received APACHE II scores < 15 and ≥15, respectively.

**Figure 1:**
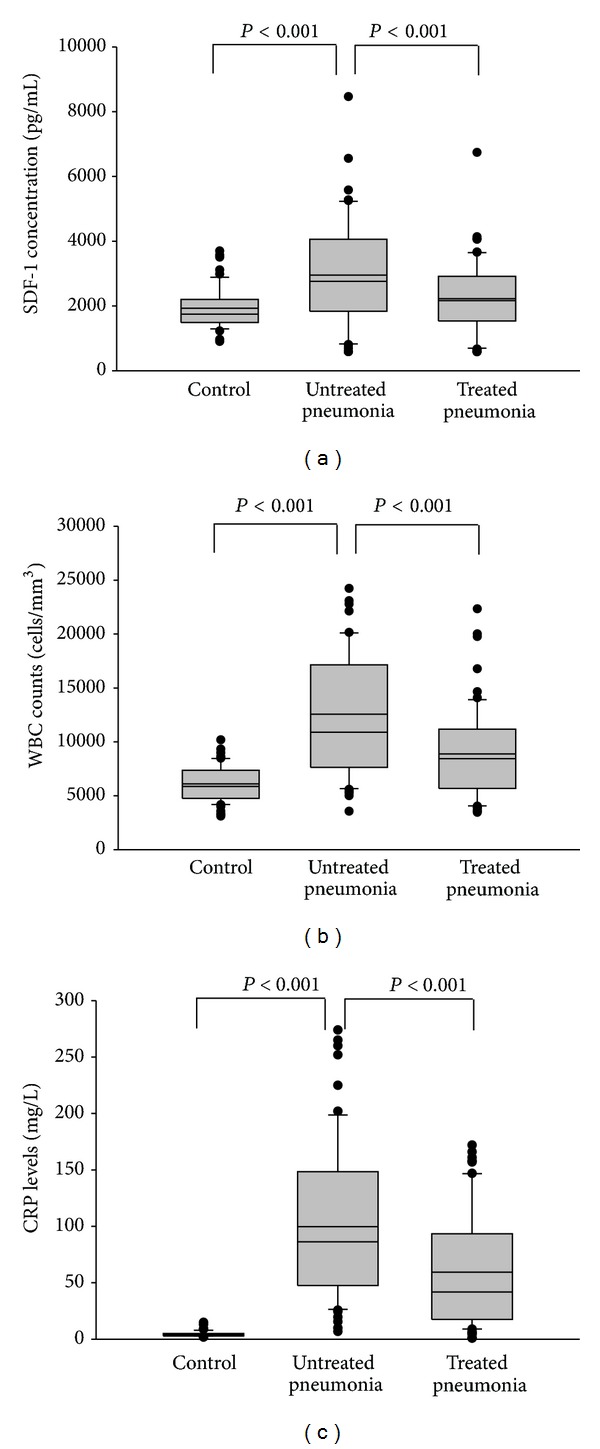
Levels of plasma (a) stromal-cell-derived factor-1 (SDF-1), (b) WBC counts, and (c) CRP levels in control subjects, and patients with community-acquired pneumonia (CAP) before and after treatment. The plasma SDF-1 level, WBC counts, and CRP levels were significantly elevated in patients with CAP before they received treatment compared to the controls and significantly decreased in CAP patients after having received treatment (*P* < 0.001).

Figure 1(a) shows the SDF-1 expression of the CAP patients and controls. A significantly increased SDF-1 level (*P* < 0.001) was observed in the plasma of patients with CAP before receiving treatment (2949 ± 1629 ng/mL) compared with healthy controls (1927 ± 625 ng/mL) and significantly decreased (*P* < 0.001) after treatment (2223 ± 1138 ng/mL). WBC, neutrophil counts, and CRP levels were significantly elevated in patients with CAP before they received treatment compared with the healthy controls (*P* < 0.001, Table 1) and patients with CAP after they received treatment (*P* < 0.001, Table 1). The WBC counts of the CAP patients before antibiotic treatment were higher than those of the controls (median 10890 versus 5860 cells/mm^3^; *P* = 0.001) (Figure 1(b)). The neutrophil counts of the CAP patients before antibiotic treatment (median 8673 versus 3530 cells/mm^3^; *P* = 0.001) were higher than those of the controls. CRP levels among CAP patients were higher than those of the controls before antibiotic treatment (median 86 versus 3 mg/L; *P* = 0.001) (Figure 1(c)). In addition, CRP levels (before antibiotic treatment: median 86 mg/L, after antibiotic treatment: median 9 mg/L; *P* = 0.001), WBC counts (before antibiotic treatment: median 10890 cells/mm^3^, after antibiotic treatment: median 8450 cells/mm^3^; *P* = 0.001), and neutrophil counts (before antibiotic treatment: median 8673 cells/mm^3^, after antibiotic treatment: median 5484 cells/mm^3^; *P* = 0.001) of patients with CAP were significantly lower after antibiotic treatment than before treatment.

**Figure 2:**
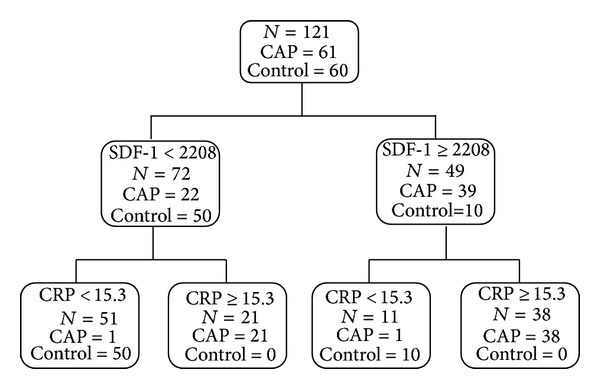
Classification and regression tree (CART) analysis for the control and CAP groups using plasma stromal-cell-derived factor-1 (SDF-1) levels and CRP data.

The cutoff levels of plasma SDF-1 and CRP were selected as 2208 ng/mL and 15.3 mg/L, respectively, according to operating characteristics curve (ROC) analysis to distinguish the patients with pneumonia from the control groups. The sensitivities of plasma SDF-1 and CRP were 63.9% and 95.1%, and the specificities were 83.3% and 96.6%, respectively. The positive predictive values of plasma SDF-1 and CRP were 79.6% and 96.6%, and the negative predictive values were 69.4% and 95.1%, respectively. For exploring the potential combination of CRP and SDF-1 in prognosticating CAP, a classification tree obtained by classification and regression tree (CART) analysis was shown in Figure 2.

**Figure 3:**
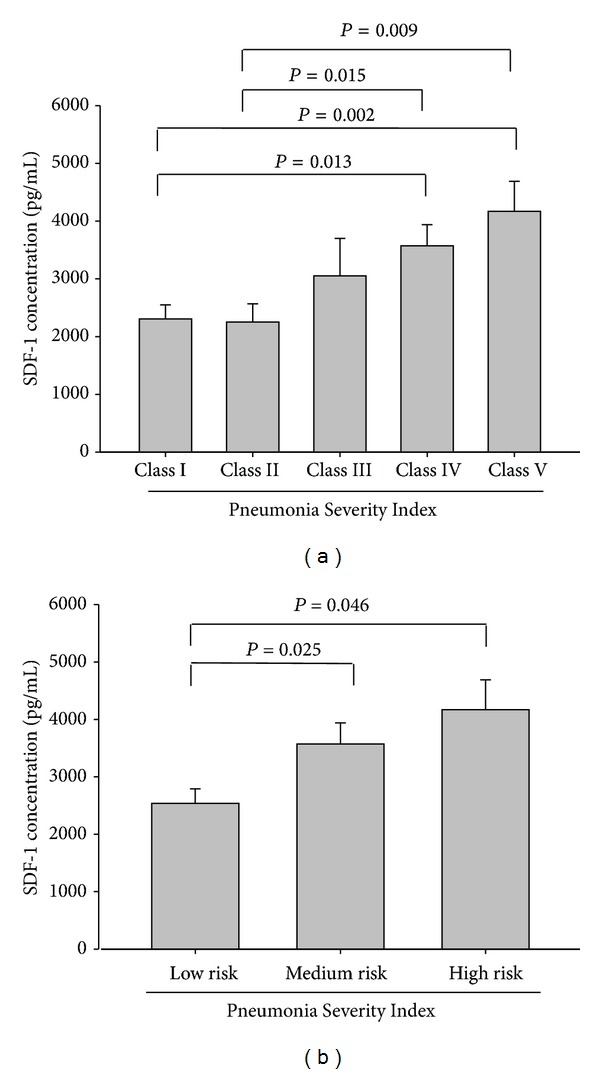
Levels of plasma stromal-cell-derived factor-1 (SDF-1) in Pneumonia Severity Index (PSI) scores in 61 patients with community-acquired pneumonia (CAP). (a) There was a significant different between Class V and Class I and Class II PSI scores. (b) There was a significant difference between high risk and low risk PSI score (*P* < 0.05).

**Figure 4:**
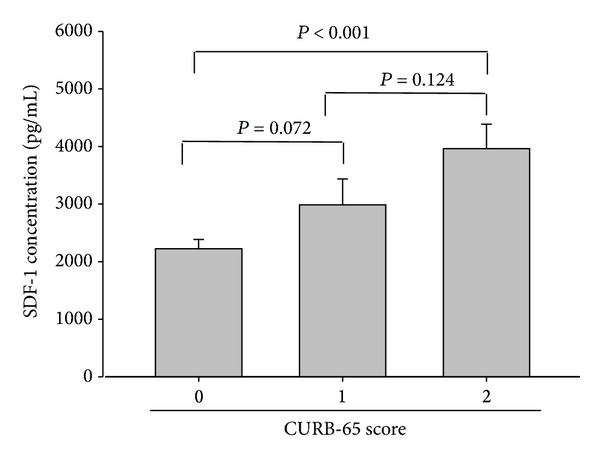
Levels of plasma stromal-cell-derived factor-1 (SDF-1) in CURB-65 scores in 61 patients with community-acquired pneumonia (CAP). There was a significant difference between patients who scored 0 and those who scored 2 (*P* < 0.001) on CURB-65.

**Figure 5:**
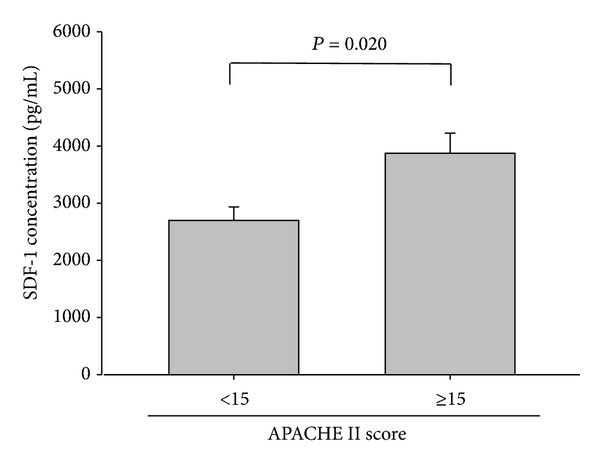
Levels of plasma stromal-cell-derived factor-1 (SDF-1) in APACHE II scores in 61 patients with community-acquired pneumonia (CAP). There was a significant difference between patients with an APACHE II score < 15 and those with a score ≥ 15 (*P* = 0.02).

Table 2 illustrates the association of the WBC count and CRP and SDF-1 levels with the PSI, CURB-65, and APACHE II scores of the CAP patients before antibiotic treatment. Neither a significant association nor a significant difference was observed between the WBC count and CRP level and the PSI (*r* = 0.005, *P* = 0.972, and *n* = 61; *r* = −0.046, *P* = 0.726, and *n* = 61), CURB-65 (*r* = 0.054, *P* = 0.677, and *n* = 61; *r* = −0.040, *P* = 0.758, and *n* = 61), and APACHE II scores (*r* = 0.063, *P* = 0.063, and *n* = 61; *r* = 0.032, *P* = 0.804, and *n* = 61). Additionally, we also found that SDF-1 levels did not exhibit a significant association with WBC, neutrophils, lymphocytes, monocytes, platelets, CRP, and the length of hospital stay (Table 2). By contrast, the SDF-1 levels of the CAP patients before receiving treatment were significantly correlated with the PSI, CURB-65, and APACHE II scores (*r* = 0.389, *P* = 0.002, and *n* = 61; *r* = 0.449, *P* < 0.001, and *n* = 61; and *r* = 0.363, *P* = 0.004, and *n* = 61, resp.). Moreover, significant differences in SDF-1 levels were observed between Class I and Class IV (*P* = 0.013), Class I and Class V (*P* = 0.002), Class II and Class IV (*P* = 0.015), and Class II and Class V (*P* = 0.009) patients (Figure 3(a)). Moreover, significantly different SDF-1 levels were observed in patients who were classified as low risk and those who were classified as medium risk (*P* = 0.025), as well as between patients who were classified as low risk and those who were classified as high risk, according to PSI scores (*P* = 0.046) (Figure 3(b)).  Figure 4 shows that the SDF-1 levels significantly differed between patients who scored 0 and those who scored 2 (*P* < 0.001) on CURB-65. Furthermore, the SDF-1 levels significantly differed between patients with an APACHE II score < 15 and those with a score ≥ 15 (*P* = 0.02) (Figure 5).

## 4. Discussion

This study showed significant differences in CRP concentrations, WBC counts, and neutrophils between patients with CAP and healthy controls. Significant differences in CRP concentrations, WBC, and neutrophil counts between patients with CAP before and after antibiotic therapy were also noted. WBC, neutrophil counts, CRP concentrations, and other biomarkers such as procalcitonin (PCT) and proadrenomedullin (pro-ADM) are used to predict the severity and prognosis of CAP [21–23]. However, the potential roles of these diagnostic markers for predicting CAP severity were still controversial [7, 24–26]. In this study, there was no significant association between CRP and any of PSI, Curb-65, or APACHE II scores. Moreover, the CRP level in CAP was affected by a different infection source. One study indicated that patients with* Legionella pneumophila* pneumonia had higher CRP levels than did those with pneumonia of any other etiology, independent of the severity of infection [27]. Elevated CRP levels were noted when patients experienced chronic inflammation caused by conditions such as cardiovascular disease, metabolic syndrome, and colorectal cancer [28]. The baseline CRP levels of patients are higher when patients are experiencing chronic inflammation. This may be one of the reasons that CRP levels are less related to PSI and CURB-65 scores than SDF-1 levels are. Patients with CAP exhibited markedly elevated CRP and SDF-1 levels, but only SDF-1 was correlated with the severity of CAP in our study, indicating that CRP levels are sensitive to pulmonary infection but are not as closely correlated with CAP severity as SDF-1. SDF-1 has been associated with inflammatory diseases such as inflammatory bowel disease, rheumatoid arthritis (RA), subacromial bursitis, and encephalitis [29–33]. Burgoyne et al. observed that the SDF-1 levels of synovial tissues were increased in RA patients who experienced relapses compared with RA patients who experienced remissions [15]. Leone et al. observed that the serum SDF-1 concentration was higher in patients with acute myocardial infarction than in healthy controls [16]. However, the relationship between SDF-1 levels and the therapeutic effect of CAP is unclear. Our results indicated a significant difference between the SDF-1 levels of patients with CAP and those of healthy controls as well as between the SDF-1 levels of patients with CAP before antibiotic treatment and after antibiotic treatment. In this study, we confirmed that the plasma concentration of SDF-1 is significantly correlated with the therapy effect for CAP patients.

The PSI is commonly used to predict the severity of CAP. The results of our study indicated a significant difference in the SDF-1 levels of patients with CAP compared with those of healthy controls as well as between the SDF-1 levels of patients with CAP before and after antibiotic treatment. We also determined that SDF-1 concentration is associated with the PSI, which is the most common index used to determine whether a patient should be hospitalized and could predict the mortality of patients with CAP. In addition, significantly different SDF-1 levels were observed between patients who were classified as low risk and those who were classified as moderate risk (*P* = 0.025) and between patients who were classified as low risk and those who were classified as high risk according to PSI scores (*P* = 0.046). Patients with CAP who are determined to be at a moderate or high risk according to the PSI should be hospitalized for treatment. According to the PSI, a moderate risk of mortality is 8.2%, and a high risk of mortality is approximately 29.2% [1]. SDF-1 is a convenient diagnostic biomarker for determining whether a patient with CAP should be hospitalized and is a valuable tool that informs physicians of the risk of mortality of patients with CAP. Moreover, a significant difference in SDF-1 levels was observed between patients with APACHE-II scores < 15 and those with scores ≥ 15 (*P* = 0.02). Our study showed that the plasma concentration of SDF-1 was significantly correlated with CURB-65, APACHE II, and, especially, PSI scores. Therefore, an elevated plasma SDF-1 concentration can be used a marker for the early detection of CAP severity. This is the first demonstration of the association between SDF-1 and CAP severity. The limitation of this study is the deficiency of microbial data. Differences in pathogens may cause differences in the severity of CAP. Therefore, our future studies will focus on the association between SDF-1 and the various microbial pathogens that cause CAP.

In conclusion, plasma SDF-1 may play a role in the clinical assessment of the severity of CAP and can potentially guide the development of treatment methods and predict clinical outcomes. Moreover, an elevated plasma SDF-1 concentration can be used as a biological marker for the early diagnosis of CAP.

## Figures and Tables

**Table 1:** Laboratory data of both controls and patients with community-acquired pneumonia (CAP) before and after they received treatment^a^.

Clinical variable	Controls (*n* = 60) Median (range)	Before antibiotic treatment (*n* = 61) Median (range)	After antibiotic treatment (*n* = 61) Median (range)	*P* value UT/C^b^	*P* value UT/T^c^
Age	59.4 ± 1.5^d^	59.5 ± 2.6		*P* = 0.963	
Gender					
Male	36 (60%)	37 (60.7%)		*P* = 0.941	
Female	24 (40%)	24 (39.3%)			
CRP (mg/L)	3 (1–17)	86 (7–274)	9 (3–113)	*P* < 0.001	*P* < 0.001
WBCs (cells/mm^3^)	5860 (3110–10190)	10890 (3560–32480)	8450 (3460–22340)	*P* < 0.001	*P* < 0.001
Neutrophils (cells/mm^3^)	3530 (1738–6046)	8673 (1032–29686)	5484 (1518–21155)	*P* < 0.001	*P* < 0.001
PSI score					
Class I		13 (21.3%)			
Class II		13 (21.3%)			
Class III		13 (21.3%)			
Class IV		18 (29.5%)			
Class V		4 (6.6%)			
CURB-65 score					
0		26 (42.6%)			
1		17 (27.9%)			
2		18 (29.5%)			
APACHE II score					
<15		48 (78.7%)			
≥15		13 (21.3%)			

CRP: C-reactive protein; WBCs: white blood cells; C: controls; UT: patients with CAP before they received antibiotic treatment; T: patients with CAP after they received antibiotic treatment.

^a^ *P* < 0.05 was considered significant.

^b^The statistical difference was analyzed by the Mann-Whitney *U* test.

^c^The statistical difference was analyzed by the Wilcoxon signed-rank test.

^d^Mean ± SD.

**Table 2:** Association of white blood cells (WBCs), C-reactive protein (CRP), and SDF-1 with clinical pathological features.

Variable	WBC (*n* = 61)		CRP (*n* = 61)		SDF-1 (*n* = 61)	
	*r*	*P* value	*r*	*P* value	*r*	*P* value
PSI score	0.005	0.972	−0.046	0.726	0.389	0.002
CURB-65 score	0.054	0.677	−0.040	0.758	0.449	<0.001
APACHE II score	0.063	0.630	0.032	0.804	0.363	0.004
Length of hospital stay	−0.044	0.736	0.086	0.183	0.183	0.157
WBC	—	—	0.130	0.319	0.241	0.061
Neutrophils	0.975	<0.001	0.138	0.289	0.247	0.055
Lymphocytes	0.251	0.051	−0.092	0.481	−0.001	0.993
Monocytes	0.672	<0.001	0.022	0.869	−0.012	0.924
Platelets	0.190	0.142	0.083	0.524	−0.008	0.954
CRP	0.130	0.319	—	—	0.082	0.528

PSI: Pneumonia Severity Index; APACHE II: Acute Physiology and Chronic Health Evaluation II.

## Abbreviations

APACHE II: Acute Physiology and Chronic Health Evaluation II
CAP: Community-acquired pneumonia
CRP: C-reactive protein
ELISA: Enzyme-linked immunosorbent assay
PSI: Pneumonia Severity Index
SDF-1: Stromal-cell-derived factor-1.

## References

[1] Lutfiyya MN, Henley E, Chang LF, Reyburn SW (2006). Diagnosis and treatment of community-acquired pneumonia. *American Family Physician*.

[2] Waterer GW, Rello J, Wunderink RG (2011). Management of community-acquired pneumonia in adults. *The American Journal of Respiratory and Critical Care Medicine*.

[3] Ramirez JA, Anzueto AR (2011). Changing needs of community-acquired pneumonia. *Journal of Antimicrobial Chemotherapy*.

[4] Department of Statistics of Ministry of Health and Welfare in Taiwan (2013). *Causes of Death in Taiwan, 2012*.

[5] Coelho L, Póvoa P, Almeida E (2007). Usefulness of C-reactive protein in monitoring the severe community-acquired pneumonia clinical course. *Critical Care*.

[6] Thiem U, Niklaus D, Sehlhoff B (2009). C-reactive protein, severity of pneumonia and mortality in elderly, hospitalised patients with community-acquired pneumonia. *Age and Ageing*.

[7] Kim JH, Seo JW, Mok JH (2013). Usefulness of plasma procalcitonin to predict severity in elderly patients with community-acquired pneumonia. *Tuberculosis and Respiratory Diseases*.

[8] Ewig S, Torres A, Woodhead M (2006). Assessment of pneumonia severity: a European perspective. *European Respiratory Journal*.

[9] Capelastegui A, España PP, Quintana JM (2006). Validation of a predictive rule for the management of community-acquired pneumonia. *European Respiratory Journal*.

[10] Richards G, Levy H, Laterre P-F (2011). CURB-65, PSI, and APACHE II to assess mortality risk in patients with severe sepsis and community acquired pneumonia in PROWESS. *Journal of Intensive Care Medicine*.

[11] Kucia M, Jankowski K, Reca R (2004). CXCR4-SDF-1 signalling, locomotion, chemotaxis and adhesion. *Journal of Molecular Histology*.

[12] Petty JM, Sueblinvong V, Lenox CC (2007). Pulmonary stromal-derived factor-1 expression and effect on neutrophil recruitment during acute lung injury. *Journal of Immunology*.

[13] Delano MJ, Kelly-Scumpia KM, Thayer TC (2011). Neutrophil mobilization from the bone marrow during polymicrobial sepsis is dependent on CXCL12 signaling. *Journal of Immunology*.

[14] Abi-Younes S, Sauty A, Mach F, Sukhova GK, Libby P, Luster AD (2000). The stromal cell-derived factor-1 chemokine is a potent platelet agonist highly expressed in atherosclerotic plaques. *Circulation Research*.

[15] Burgoyne CH, Field SL, Brown AK (2008). Abnormal T cell differentiation persists in patients with rheumatoid arthritis in clinical remission and predicts relapse. *Annals of the Rheumatic Diseases*.

[16] Leone AM, Rutella S, Bonanno G (2006). Endogenous G-CSF and CD34^+^ cell mobilization after acute myocardial infarction. *International Journal of Cardiology*.

[17] Mandell LA, Wunderink RG, Anzueto A (2007). Infectious Diseases Society of America/American Thoracic Society Consensus Guidelines on the management of community-acquired pneumonia in adults. *Clinical Infectious Diseases*.

[18] Fine MJ, Auble TE, Yealy DM (1997). A prediction rule to identify low-risk patients with community-acquired pneumonia. *The New England Journal of Medicine*.

[19] Knaus WA, Draper EA, Wagner DP, Zimmerman JE (1985). APACHE II: a severity of disease classification system. *Critical Care Medicine*.

[20] Lim WS, Van Der Eerden MM, Laing R (2003). Defining community acquired pneumonia severity on presentation to hospital: an international derivation and validation study. *Thorax*.

[21] Chalmers JD, Singanayagam A, Hill AT (2008). C-reactive protein is an independent predictor of severity in community-acquired pneumonia. *The American Journal of Medicine*.

[22] Smith RP, Lipworth BJ (1995). C-reactive protein in simple community-acquired pneumonia. *Chest*.

[23] Torres A, Ramirez P, Montull B, Menéndez R (2012). Biomarkers and community-acquired pneumonia: tailoring management with biological data. *Seminars in Respiratory and Critical Care Medicine*.

[24] de Jager CPC, Wever PC, Gemen EFA (2012). The neutrophil-lymphocyte count ratio in patients with community-acquired pneumonia. *PLoS ONE*.

[25] Christ-Crain M, Morgenthaler NG, Stolz D (2006). Pro-adrenomedullin to predict severity and outcome in community-acquired pneumonia. *Critical Care*.

[26] Boussekey N, Leroy O, Georges H, Devos P, D'Escrivan T, Guery B (2005). Diagnostic and prognostic values of admission procalcitonin levels in community-acquired pneumonia in an intensive care unit. *Infection*.

[27] García Vázquez E, Martínez JA, Mensa J (2003). C-reactive protein levels in community-acquired pneumonia. *European Respiratory Journal*.

[28] Black S, Kushner I, Samols D (2004). C-reactive protein. *The Journal of Biological Chemistry*.

[29] Werner L, Guzner-Gur H, Dotan I (2013). Involvement of CXCR4/CXCR7/CXCL12 interactions in inflammatory bowel disease. *Theranostics*.

[30] Charo IF, Ransohoff RM (2006). Mechanisms of disease: the many roles of chemokines and chemokine receptors in inflammation. *The New England Journal of Medicine*.

[31] Kim Y-S, Bigliani LU, Fujisawa M (2006). Stromal cell-derived factor 1 (SDF-1, CXCL12) is increased in subacromial bursitis and downregulated by steroid and nonsteroidal anti-inflammatory agents. *Journal of Orthopaedic Research*.

[32] Sánchez-Martín L, Sánchez-Mateos P, Cabañas C (2013). CXCR7 impact on CXCL12 biology and disease. *Trends in Molecular Medicine*.

[33] Tsai H-T, Tee Y-T, Hsieh Y-H (2009). Elevated plasma stromal cell-derived factor 1 protein and its gene polymorphism in patients with pelvic inflammatory disease. *Reproductive Sciences*.

